# The effects of heat-moisture treatment on resistant starch levels in cassava and on fermentation, methanogenesis, and microbial populations in ruminants

**DOI:** 10.14202/vetworld.2023.811-819

**Published:** 2023-04-20

**Authors:** Legi Okta Putra, Sri Suharti, Ki Ageng Sarwono, Sutikno Sutikno, Ainissya Fitri, Wulansih Dwi Astuti, Rohmatussolihat Rohmatussolihat, Yantyati Widyastuti, Roni Ridwan, Rusli Fidriyanto, Komang Gede Wiryawan

**Affiliations:** 1 Study Program of Nutrition and Feed Science, Graduate School of IPB University, Bogor, Indonesia; 2 Department of Nutrition and Feed Technology, IPB University, Bogor, Indonesia; 3 Research Center for Applied Zoology, National Research and Innovation Agency (BRIN), Cibinong, Indonesia

**Keywords:** heat-moisture treatment, *in vitro*, rumen fermentation, starch modification

## Abstract

**Background and Aim:**

Resistant starch (RS) is difficult to digest in the digestive tract. This study aimed to evaluate the effects of heat-moisture treatment (HMT) on RS in cassava and examined its impact on rumen fermentation.

**Materials and Methods:**

Cassava flour was used as a raw material and used in a randomized block design with four different cycles of HMT as the treatments and four different rumen incubations *in vitro* as blocks. Treatments included: HMT0: without HMT (control), HMT1: one HMT cycle, HMT2: two HMT cycles, and HMT3: three HMT cycles. Heat-moisture treatment processes were performed at 121°C for 15 min and then freezing at −20°C for 6 h. Analyzed HMT cassava starch characteristics included components, digestibility, and physicochemical properties. In *in vitro* rumen fermentation studies (48 h incubation) using HMT cassava, digestibility, gas production, methane, fermentation profiles, and microbial population assessments were performed.

**Results:**

Heat-moisture treatment significantly reduced (p < 0.05) starch, amylopectin, rapidly digestible starch (RDS), and slowly digestible starch levels. In contrast, amylose, reducing sugars, very RDS, RS, and protein digestion levels were significantly increased (p < 0.05). Additionally, a reduced crystallinity index and an increased amorphous index were observed in starch using Fourier-transform infrared analyses, while a change in crystalline type from type A to type B, along with a reduction in crystallinity degree, was observed in X-ray diffraction analyses. Heat-moisture treatment significantly (p < 0.05) reduced rumen dry matter (DM) degradation, gas production, methane (CH_4_ for 12 h), volatile fatty acid (VFA), and propionate levels. In addition, acetate, butyrate, and acetate/propionate ratios, as well as population of *Streptococcus* *bovis* and *Bacteroides* were significantly increased (p < 0.05). However, pH, ammonia, and organic matter digestibility were unaffected (p > 0.05) by HMT.

**Conclusion:**

Cassava HMT altered starch characteristics, significantly increased RS, which appeared to limit rumen digestion activity, decreased rumen DM degradation, gas production, VFAs, and CH_4_ production for 12 h, but increased *S. bovis* and *Bacteroides* levels.

## Introduction

In high-performance and intensive farming, dairy or beef cattle are fed high-energy diets to maximize productivity and economic efficiency [1]. Diets usually contain easily degradable plant carbohydrates such as starch. Higher dietary starch concentrations encourage rapid microbial growth in the rumen and volatile fatty acid (VFA) formation, which are valuable energy sources as starch provides immediately accessible glucose for microbes [2]. Starch digestion in ruminants occurs in the rumen, post-rumen, and intestines. In the rumen, starch digestion rapidly produces VFAs [3] as starch is an easily digestible carbohydrate and VFAs are primary energy sources for livestock and rumen microbes. Starch digestion in the rumen also produces carbon dioxide (CO_2_) and hydrogen (H_2_) substrates for methane (CH_4_) production [4]. Starch digestion also occurs in post-rumen digestive organs and intestines. Intestinal enzymes from the pancreas, including amylase, maltase, and isomaltase, digest starch to glucose which is absorbed [2]. Fidriyanto *et al*. [5] reported that energy production in the post-rumen was more efficient when compared with the rumen as starch fermentation byproducts in the rumen were transformed to heat and CH_4_. Cerrilla and Martinez observed that ruminants digested large amounts of starch in the small intestine [2].

Apart from the importance of starch to ruminants, high starch levels may be dangerous to due to rapid VFA production, leading to reduced pH levels in rumen fluids [6]. This may lead to ruminant digestive disorders, such as subacute rumen acidosis or acute ruminant acidosis [3]. Low pH conditions may also inhibit cellulolytic bacterial populations, affecting fiber and dry matter (DM) intake. Starch digestion in the rumen is also accompanied by energy losses through heat and CH_4_ [7]. Thus, strategies are required to limit starch digestion in the rumen and maximize its post-rumen digestion effects. According to a previous study [8], modifying starch-containing feed resources using heat-moisture treatment (HMT) could be used to modify starch digestion sites in ruminants, from digestion at the rumen to post-rumen sites. In ruminants, starch is an indispensable energy source that maximizes production capabilities. However, high starch levels may cause digestive disorders due to decreased pH levels in rumen fluids from high VFA levels [6]. To overcome these issues, rumen-resistant starch (RS) can be used to limit starch digestion in the rumen and maximized the output of starch digestion in post-rumen organs. In a previous study [8], HMT was used to modify starch-containing feed sources to increase RS levels in livestock.

Heat-moisture treatment is a promising starch-processing method due to its straightforward heating and freezing steps. From temperature-water interactions, some of the starch polymerizes to form colloidal air solutions or starch gels or paste. Then, helical chains crystallize during low-temperature storage to produce resistant starch type 3 (RS3), which resists amylolytic enzyme activity [9]. However, HMT-treated cassava and RS effects on rumen fermentation, methanogenesis, and rumen microbial populations remain unclear.

Therefore, this study aimed to investigate the HMT effects on cassava nutrition and physicochemical starch characteristics, and examined rumen digestibility, methanogenesis, and rumen microbial populations *in vitro*.

## Materials and Methods

### Ethical approval

The protocol for animal handling was approved by the Animal Ethics Committee of the Indonesian Institutes of Sciences (approval number 39/klirens/III/2021).

### Study period and location

The study was conducted from September 2021 to June 2022 at the Laboratory of Nutrition and Feed Biotechnology, Research Center for Biotechnology, National Research and Innovation Agency (BRIN) in Cibinong, West Java, Indonesia.

### Sample preparation

Tuber of cassava (Manihot esculenta) samples were collected from farmers in Sukabumi, West Java, Indonesia. The plant was identified by Ki Ageng Sarwono (Research Center for Applied Zoology, National Research, and Innovation Agency) according to the description by Fukuda *et al*. [10]. The tubers were ground into flour and processed according to Berry [11] with some modifications. Flour cassava samples were mixed with distilled water (1:3.5 w/v) and underwent HMT cycles, which consisted of 121°C for 15 min and then cooling to −20°C for 6 h. Samples were subjected to 0, 1, 2, and 3 HMT cycles (4 in total).

### Proximate and total starch analysis

Analyses were performed according to the Association of Official Analytical Chemists (AOAC) [12] and included DM, organic matter (OM), crude fat, crude fiber, and crude protein measurements. The total starch content in cassava flour was analyzed using Megazyme total starch kit (Neogen, USA) following manufacturer instructions.

### Amylose and amylopectin levels

Amylose and amylopectin levels were analyzed according to Williams *et al*. [13]. Samples (0.1 g) were mixed with 1 M NaOH (9 mL) and ethanol (1 mL) and then boiled for 10 min. After sample temperature was the same as room temperature (28°C), samples were transferred to a 100 mL standard flask and distilled water was added to 100 mL. Then, 5 mL samples were mixed with 1 mL acetic acid (1 M) and 2 mL iodine solution in another 100 mL volumetric flask and distilled water was added to the 100 mL mark. Absorbance was recorded using a spectrophotometer at 620 nm and calculated with the formula below.


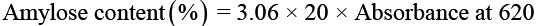


### Very rapidly digestible starch (VRDS), rapidly digestible starch (RDS), slowly digestible starch (SDS), and RS quantification

Procedures were adapted from Sopade and Gidley [14] and glucose concentrations were recorded using a spectrophotometer (Ultraviolet-visible, Shimadzu, 1800, Japan). Samples (0.5 g) were mixed with 1 mL artificial saliva and 5 mL pepsin in 0.02M hydrochloric acid (HCl) (pH 2.0) and incubated at 37°C for 30 min at 85 rpm. Digests were neutralized in 5 mL NaOH (0.02 M) and the pH was adjusted to 6 before adding 5 mL pancreatin and amyloglucosidase solutions. Samples were incubated at 37°C at 85 rpm for 0 (VRDS), 30 (RDS), and 120 (SDS) min, and then 1 mL was removed and mixed with 9 mL absolute ethanol. Then, 1 mL was boiled with 1 mL DNS for 10 min and glucose concentrations were recorded using a spectrophotometer at 540 nm. The content of VRDS, RDS, SDS, and RS was determined by multiplying respective starch’s glucose concentration by the total starch content.

### Fourier-transform infrared (FTIR) analysis

Sample functional groups were analyzed using FTIR spectrometry (Perkin-Elmer UATR Two, USA). Samples were ground and filtered through a 60 mesh prior to analysis. Spectra were recorded in the 4000–400 cm^−1^ range at a resolution of 4 cm^−1^, with 1 cm^−1^ intervals. Crystalline and amorphous index (CI and AI, respectively) were determined as the ratio of intensities at certain wavenumbers (cm^−1^) as: 1047/1022, and 1022/995, respectively [15].

### X-ray diffraction (XRD)

Samples were prepared for XRD analysis using a 100 mesh. Diffraction scanning (XPert PRO, Panalytical, Netherlands), using Cu radiation, ranged from 10° to 80° 2θ with a size of 0.02, current = 30 mA, voltage = 40 kV, and scan speed = 2° min^−1^. Percent of crystallinity was calculated using the equation below.


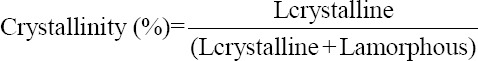


### *In vitro* rumen fermentation

Rumen fluid was collected from four male fistulated cattle (Ongole crossbreed) which were kept in separate cages. The rumen incubation *in vitr*o was conducted following the procedure of Theodorou *et al*. [16]. Before morning feeding, rumen fluid was collected through the fistula, filtered into a thermos flask (39°C) through four cheesecloth layers, and transported to the laboratory. Filtered rumen fluid and flushed McDougall buffer (using CO_2_ gas) were mixed at a 1:2 ratio at 39°C and added to a serum bottle (100 mL) which contained 0.5 g finely ground sample (substrate). Before sealing, CO_2_ was flushed into bottles for 30 s to maintain anaerobic conditions. The bottles were sealed using rubber septum and aluminum crimp and incubated at 39°C for 48 h. Samples were duplicated for rumen and post-rumen digestibility analyses.

During incubation, gas was collected at 2, 4, 6, 8, 10, 12, 24, and 48 h using a 50 mL syringe to measure gas production and kinetics. Methane emissions at 12 h, 24 h, and 48 h were recorded using an infrared methane analyzer (Riken RX-415, Japan). After 48 h, samples were centrifuged at 5080× *g* for 10 min and supernatants were processed to measure pH, ammonia-N (NH_3_–N), total VFAs, and microbial populations. Filtered residues (Whatman filter paper No. 41) were used for DM and OM degradation analyses. Sediments from other replicates were incubated for 48 h in 50 mL pepsin–HCl solution and filtered through Whatman paper No. 4. for digestion analysis. To analyze DM and OM, filtered samples were processed as previously described by AOAC [12].

### DNA extraction

Microbial DNA was extracted from rumen fluid supernatants following QIAamp DNA Stool Mini Kit (Qiagen, Hilden, Germany) instructions. Extracted DNA was stored at −30°C until further analysis.

### Quantifying rumen microbial populations using quantitative real-time polymerase chain reaction (qRT-PCR)

Extracted DNA was used to the cycle threshold (C_T_) of rumen microbial populations, including the methanogens *Butyrivibrio fibrisolvens*, *Streptococcus* *bovis*, *Bacteroides*, and *Selonomonas* *ruminantium* using real-time PCR. The relative quantification method used was comparative 2^−ΔΔCT^ method according to Schmittgen and Livak [17]. Specific primers of the target group and the amount used for real-time PCR are listed in Table-1 [18]. In the calculation of 2^−ΔΔCT^ value, C_T_ of total bacteria were used as an endogenous control. Reaction mixtures (20 μL) consisted of DNA, SYBR qPCR master mix, 50× ROX (Qiagen, Hilden, Germany), forward (f) and reverse (r) primers, and sterile distilled water. Polymerase chain reaction conditions were: One cycle at 95°C for 1 min, 40 denaturing cycles at 95°C for 15 s, and annealing and extension at 60°C for 1 min, except for methanogens which required 60°C for 30 s.

**Table-1:** Primers used for polymerase chain reaction [18].

Target	Name	Sequence (5’- 3’)	Amount added (m)
Total bacteria	1114-f 1275-r	CGGCAACGAGCGCAACCC CCATTGTAGCACGTGTGTAGCC	0.6 0.6
Methanogen	q-mcrA-f q-mcrA-r	TTCGGTGGATCDCARAGRGC GBARGTCGWAWCCGTAGAATCC	1.2 1.2
Genus *Bacteroides*	AllBac 296-f AllBac 412-r	GAGAGGAAGGTCCCCCAC CGCTACTTGGCTGGTTCAG	0.2 3.6
*Selonomonas* *ruminantium*	SelRum 2F SelRum 2R	CAATAAGCATTCCGCCTGGG TTCACTCAATGTCAAGCCCTGG	0.45 0.45
*Butyvibrio* *fibrisolvens*	ButFib 2F ButFib 2R	ACCGCATAAGCGCACGGA CGGGTCCATCTTGTACCGATAAAT	0.2 0.2
*Streptococcus* *bovis*	StrBov 2F StrBoy 2R	TTCCTAGAGATAGGAAGTTTCTTCGG ATGATGGCAACTAACAATAGGGGT	8.8 8.8

### Statistical analysis

A randomized complete block design was used in this study. Data analysis was conducted using a one-way analysis of variance (ANOVA). Different rumen incubation *in vitr*o run was used as the blocks in ANOVA statistical model. The model of statistical in this study follows arranged:


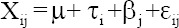


Where X_ij_ is the observed value; μ is the grand mean; τ_i_ is the effect of the treatment; β_j_ is the block effect; ɛ_ij_ is the random error. Significant difference in results were accepted at the probability level of p < 0.05 and then was carried out for Tukey’s test. The data were analyzed using the statistical app of statistical package for the social sciences (SPSS) version 26.0 (SPSS Inc., Chicago, IL, USA).

The calculation of gas production kinetics followed the equation of López *et al*. [19]:


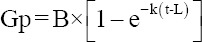


Where Gp: Gas production for 48 h (mL), B: Maximum production of gas (mL/g), L: Lag time (h), k: Rate production of gas (mL/h), and t: The incubation time (h).

## Results

### Nutrient characteristics

Sample nutrient characteristics were significantly (p < 0.01) affected by HMT (Table-2). HMT increased DM, crude protein, and crude fat levels, while OM and crude fiber levels decreased. The highest DM content was at two cycles of HMT (HMT2), while the lowest was observed at HMT0. The highest crude protein content was recorded at one cycle of HMT (HMT1), while the highest crude fat rate was at three cycles of HMT (HMT3). Organic matter content percentages across all treatments were lower when compared with the control. The crude fiber content was also lower in HMT samples when compared with the control.

**Table-2:** Nutrient content of cassava-treated HMT with different cycles.

Parameter	HMT0	HMT1	HMT2	HMT3	SEM	p-value
Dry matter (%)	85.80^d^	88.57^c^	90.75^a^	89.55^b^	0.481	<0.001
Organic matter (% DM)	97.57^a^	96.90^b^	96.95^b^	96.94^b^	0.077	<0.001
Crude protein (% DM)	1.25^c^	2.14^a^	2.04^ab^	1.94^b^	0.091	<0.001
Crude fat (% DM)	0.45^b^	0.53^b^	0.93^b^	3.41^a^	0.320	<0.001
Crude fiber (% DM)	5.92^a^	3.78^b^	3.64^b^	3.33^b^	0.339	0.008

HMT=Heat-moisture treatment, HMT0=Without heat-moisture treatment (control), HMT1=One cycle of heat-moisture treatment, HMT2=Two cycles of heat-moisture treatment, HMT3=Three cycles of heat-moisture treatment, SEM=Standard error of mean, DM=Dry matter, and superscript differences on the same line indicate significant differences (p < 0.05)

### Physicochemical and starch characteristics

HMT increased amylose (p = 0.001) and reduced sugar levels (p < 0.001). The amylose content was increased by HMT at the expense of amylopectin, with the highest amylose content observed at HMT3. Higher HMT increased reducing sugar percentages, with the highest at HMT3. Furthermore, starch content was significantly decreased by HMT2 and HMT3.

*In vitro* starch digestion analyses showed that HMT strongly affected starch classification (p < 0.001) (Table-3). Very rapidly digestible starch and RS levels in HMT samples were higher when compared with the control. Furthermore, RDS and SDS values decreased, except for HMT3 SDS treatments, which were not significantly different to HMT0. Protein digestibility analyses showed that HMT1 and HMT2 increased the protein digestibility of starch *in vitro*.

**Table-3:** Physicochemical characteristics of cassava HMT with different cycles.

Parameter	HMT0	HMT1	HMT2	HMT3	SEM	p-value
Total starch (%)	79.10^a^	77.76^a^	71.27^b^	67.49^b^	1.337	<0.001
Amylose (% TS)	23.14^c^	24.06^bc^	26.83^ab^	28.07^a^	0.600	0.001
Amylopectin (% TS)	55.96^a^	53.69^a^	44.45^b^	39.42^b^	1.911	<0.001
Reducing sugar (mg/g)	2.69^c^	3.10^c^	6.12^b^	7.44^a^	2.607	<0.001
*In vitro* starch digestion						
VRDS (% DM Starch)	4.69^c^	7.65^b^	9.76^a^	8.36^b^	0.490	<0.001
RDS (% DM Starch)	14.84^a^	9.42^b^	8.00^c^	6.57^d^	0.820	<0.001
SDS (% DM Starch)	8.41^a^	6.72^b^	5.48^c^	8.12^a^	0.322	<0.001
RS (% DM Starch)	6.70^d^	10.59^c^	13.86^b^	17.56^a^	1.038	<0.001
Protein digestibility (%)	72.31^b^	80.20^a^	79.20^a^	74.64^b^	0.962	0.001
FTIR						
Crystalline Index (1045/1022)	0.712	0.689	0.691	0.682		
Amorphous Index (1022/995)	0.910	0.983	0.981	0.962		
XRD						
Degree of Crystallinity (%)	25.25	15.92	15.5	14.14		

HMT=Heat-moisture treatment, HMT0=Without heat-moisture treatment (control), HMT1=One cycle of heat-moisture treatment, HMT2=Two cycles of heat-moisture treatment, HMT3=Three cycles of heat-moisture treatment, SEM=Standard error of mean, TS=Total starch, VRDS=Very rapidly digested starch, RDS=Rapidly digested starch, SDS=Slowly digested starch, RS=Resistant starch, DM=Dry matter, FTIR=Fourier-transform infrared spectroscopy, XRD=X-ray diffraction, and superscript differences on the same line indicate highly significant differences (p < 0.01)

### Fourier-transform infrared and XRD analysis

**Figure-1:**
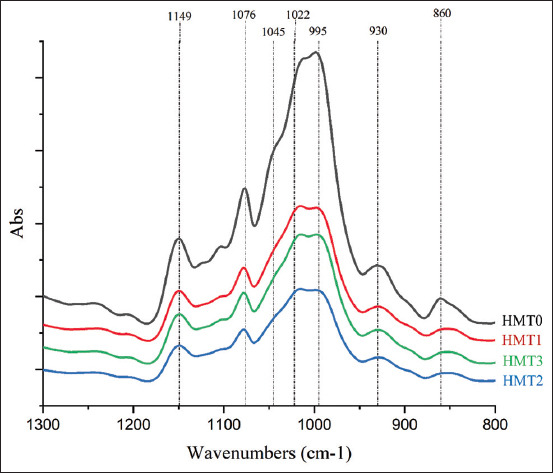
Spectra of Fourier-transform infrared of cassava heat-moisture treatment in the wavenumber rang 1300 cm^−^–800 cm^−1^, HMT0=Without heat-moisture treatment (control), HMT1=One cycle of heat-moisture treatment, HMT2=Two cycles of heat-moisture treatment, and HMT3=Three cycles of heat-moisture treatment.

Fourier-transform infrared absorbance values in the 1200–800 cm^-1^ band range were sensitive to starch composition and structure changes (Figure-1). CI and AI data are shown (Table-3). HMT reduced the CI when compared with the control (HMT0); the lowest CI value was identified in HMT3, then HMT1 and HMT2. However, AI values increased; the highest value was recorded in HMT1, then HMT2 and HMT3.

**Figure-2:**
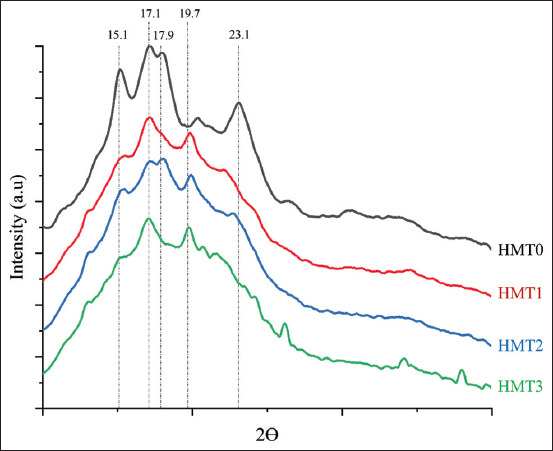
Spectra of X-ray diffraction of cassava heat-moisture treatment in the range 2Ɵ 10–40°, HMT0=Without heat-moisture treatment (control), HMT1=One cycle of heat-moisture treatment, HMT2=Two cycles of heat-moisture treatment, and HMT3=Three cycles of heat-moisture treatment.

The degree of starch crystallinity (calculated from XRD patterns) was affected by HMT (Table-3). Crystallinity degree percentages in HMT samples were relatively lower when compared with the control: The lowest value was recorded for HMT3, then HMT2 and HMT1. X-ray diffraction showed strong peak 2Ɵ patterns at 15°, 17°, 18°, and 23° for HMT0, while HMT1, HMT2, and HMT3 samples displayed firm peaks at 17° and/or 18° (Figure-2).

### Rumen digestibility and fermentation profiles *in vitro*

Rumen digestibility analyses showed that HMT decreased rumen DM degradation (DMD) (p < 0.05; Table-4), but post-rumen DMD and DM digestibility were not significantly affected. HMT0 had the highest rumen DMD levels, while HMT1 and HMT2 had the lowest. Treatments did not affect rumen OM degradation (OMD), post-rumen OMD, and OM digestibility.

**Table-4:** *In vitro* rumen digestibility of HMT-treated cassava with different cycles for 48 h incubation.

Parameter	HMT0	HMT1	HMT2	HMT3	SEM	p-value
Rumen DMD (%)	85.62^a^	81.52^b^	81.99^b^	83.02^ab^	1.239	0.011
Post-rumen DMD (%)	4.88	5.55	4.68	5.55	1.693	0.980
DM digestibility (%)	90.50	87.06	86.66	88.56	0.881	0.335
Rumen OMD (%)	97.54	96.01	96.38	96.80	0.526	0.237
Post-rumen OMD (%)	2.04	2.71	2.90	2.57	0.609	0.767
OM digestibility (%)	99.57	98.71	99.28	99.37	0.189	0.208

HMT=Heat-moisture treatment, HMT0=Without heat-moisture treatment (control), HMT1=One cycle of heat-moisture treatment, HMT2=Two cycles of heat-moisture treatment, HMT3=Three cycles of heat-moisture treatment, SEM=Standard error of mean, DM=Dry matter, OM=Organic matter, DMD=Dry matter degradation, OMD=Organic matter degradation, and superscript differences on the same line indicate significant differences (p < 0.05)

Heat-moisture treatment significantly (p < 0.01) decreased gas production at 48 h (Table-5). The lowest gas production was observed in HMT2. Heat-moisture treatment increased gas production at 2 h, 4 h, and 6 h. HMT2 recorded the highest gas production at 2 h, while HMT1 had the highest gas production at 4 h and 6 h. However, at 24 h–48 h, significant decreases in gas production were recorded (p < 0.05), with the lowest decline at HMT2. Gas production calculations using equations from López *et al*. [18] identified reductions in maximum gas production (B), but gas production rates (k) and time lags (L) were significantly increased (p < 0.05) by HMT. Methane concentrations were affected during initial 12 h incubations (p < 0.01), but gas production at 24 h–48 h showed no significant effects.

**Table-5:** Gas production kinetics and methane concentrations of different HMT-treated cassava samples.

Parameter	HMT0	HMT1	HMT2	HMT3	SEM	p-value
Gas production						
2 h (mL)	0.75^b^	3.75^ab^	4.25^a^	2.88^ab^	0.630	0.041
4 h (mL)	6.13^b^	18.00^a^	18.75^a^	16.50^a^	1.702	<0.001
6 h (mL)	19.00^b^	31.38^a^	29.88^a^	27.00^a^	1.848	0.002
8 h (mL)	35.00	40.63	37.75	36.75	1.369	0.098
10 h (mL)	47.50	48.63	44.13	43.13	1.693	0.176
12 h (mL)	59.75	55.63	52.13	52.38	1.706	0.042
24 h (mL)	86.13^a^	79.50^ab^	72.25^b^	75.38^ab^	2.993	0.025
48 h (mL)	102.75^a^	98.38^ab^	88.50^b^	92.63^b^	3.050	0.007
B (mL/g)	119.32^a^	91.36^b^	95.72^b^	87.21^b^	3.724	<0.001
k (mL/h)	0.06^b^	0.07^b^	0.09^a^	0.09^a^	0.004	<0.001
L (h)	1.45^b^	2.11^a^	1.54^b^	1.67^b^	0.161	0.002
CH_4_ 12 h (mL)	2.31^a^	0.92^b^	1.42^ab^	1.31^b^	0.162	0.007
CH_4_ 24 h (mL)	4.66	3.91	3.02	4.18	0.639	0.475
CH_4_ 48 h (mL)	6.06	6.65	5.26	6.85	0.292	0.289

HMT=Heat-moisture treatment, HMT0=Without heat-moisture treatment (control), HMT1=One cycle of heat-moisture treatment, HMT2=Two cycles of heat-moisture treatment, HMT3=Three cycles of heat-moisture treatment, SEM=standard error of mean, *B*=Maximum gas production, *k*=Rate of gas production, *L*=Lag time, h=Hour, and superscript differences on the same line indicate significant differences (p < 0.05)

Rumen fermentation profiles were affected by HMT (Table-6). No significant differences in pH and NH_3_–N levels were identified. Heat-moisture treatment treatments significantly decreased total VFA (p < 0.001) and propionate production (p < 0.01), while acetate (p < 0.05) and acetate/propionate (A/P) ratio (p < 0.01) levels increased. Three HMT cycles recorded the lowest total VFA levels but had the highest acetate, propionate, and A/P ratio values.

**Table-6:** *In vitro* rumen fermentation profile of cassava-treated with different HMT cycles in 48 h incubation.

Parameter	HMT0	HMT1	HMT2	HMT3	SEM	p-value
pH	6.95	6.93	6.98	6.96	0.007	0.110
NH_3_-N (mg/dL)	3.89	3.50	2.98	2.86	0.198	0.059
Total VFA (mmol/dL)	82.01^a^	72.98^b^	71.37^b^	68.97^c^	1.303	<0.001
Acetate (% Total VFA)	56.82^b^	59.71^a^	58.17^ab^	59.45^a^	0.376	0.010
Propionate (% Total VFA)	30.50^a^	27.02^b^	28.23^b^	26.88^b^	0.484	0.001
Butyrate (% Total VFA)	9.12^b^	9.76^ab^	9.85^ab^	10.00^a^	0.184	0.026
A/P Ratio	1.86^b^	2.22^a^	2.06^ab^	2.22^a^	0.048	0.002

HMT=Heat-moisture treatment, HMT0=Without heat-moisture treatment (control), HMT1=One cycle of heat-moisture treatment, HMT2=Two cycles of heat-moisture treatment, HMT3=Three cycles of heat-moisture treatment, SEM=Standard error of mean, VFA=Volatile fatty acid, A/P=Acetate/propionate, and superscript differences on the same line indicate significant differences (p < 0.05)

### Rumen microbial populations

Heat-moisture treatment did not significantly affect (p > 0.05) the relative quantity of methanogens, *B. fibrisolvens* and *S. ruminantium* (Table-7). However, HMT significantly (p < 0.05) increased the relative quantity of *S. bovis* and *Bacteroides genera*.

**Table-7:** Relative density of microbial populations with 2^−ΔΔCT^ of different HMT-treated cassava samples in 48 h incubation.

Parameter	HMT0	HMT1	HMT2	HMT3	SEM	p-value
Methanogen	1.06	0.65	0.63	0.75	0.100	0.321
*Butyrivibrio fibrisolvens*	1.08	0.97	1.24	1.38	0.123	0.223
*Streptococcus bovis*	1.18^b^	6.42^a^	6.29^a^	6.71^a^	0.630	<0.001
Genus *Bacteroides*	1.01^b^	3.00^a^	3.09^a^	3.55^a^	0.315	0.007
*Selenomonas ruminantium*	1.22	1.64	2.12	1.80	0.150	0.119

HMT=Heat-moisture treatment, HMT0=Without heat-moisture treatment (control), HMT1=One cycle of heat-moisture treatment, HMT2=Two cycles of heat-moisture treatment, HMT3=Three cycles of heat-moisture treatment, SEM=Standard error of mean, and superscript differences on the same line indicate significant differences (p < 0.05)

## Discussion

Cassava is abundant in the tropics and is frequently used as a starch-rich feedstuff for high-performance cattle farms. Our finding showed that local cassava from Indonesia had a high starch content of 79% (Table-3). Starch in cassava root is the main starch compound and accounts for 80%–90% of the root’s dry weight [20].

It was observed that HMT altered nutrition characteristics in cassava, in agreement with previous findings identifying characteristic nutritional changes in different starch sources after HMT [21]. Yang *et al*. [22] reported that HMT lowered moisture, crude fiber, ash, crude protein, and crude fat levels. This finding was in contrast with our findings of decreased OM and increased crude protein and fat content by HMT. However, Babu and Parimalavalli [23] and Faridah and Silitonga [24] reported an increase in cassava crude protein content caused by HMT treatment, which is similar with the finding in our study. The increase in protein content might be caused by high autoclaving temperature, which might unbind the bound protein in the starch granule and increase the crude protein content in cassava.

HMT exerted significant effects on starch characteristics in cassava; the process decreased total starch content, and then increased reducing sugar and amylose content. Simanjuntak *et al*. [25] showed that reducing sugars were increased during heating. Starch (polysaccharide), which is mainly composed of glucose (monosaccharide), was degraded at higher temperatures [26]. Reducing sugars, which mainly include fructose, glucose, lactose, and maltose, are comprised of monosaccharides or disaccharides [27]. In addition, HMT also affected amylose and amylopectin content in cassava; amylose content increased at the expense of amylopectin after HMT. A previous study reported that high HMT temperatures broke amylopectin-branched chains at 1–6 glycoside bonds, leading to linear double helix short chains, or amylose [28].

In terms of its enzymatic digestibility rates, starch is characterized into VRDS, RDS, SDS, and RS fractions [29, 30]. In our study, HMT changed starch characteristics and proportions in cassava; it shifted main RDS starch components to RS. Then the higher HMT cycles led to higher RS content. This finding concurred with the previous findings; several HMT cycles increased RS yields in the sample [31] because amylose was rapidly crystalized and formed RS3 [24].

Our result also showed that increasing RS decreased RDS, similar to Chung *et al*. [32]. Interestingly, HMT also increased VRDS content in cassava, and was possibly related to increase reduced sugar content. Reducing sugars are simple carbohydrates that are more easily digested by enzymes, thus increasing VRDS levels. Furthermore, HMT increased protein digestibility as the process increased crude protein levels; it is consistent with the study by Xia *et al*. [33], which showed that protein digestion increased linearly with increased crude protein levels.

FTIR showed that HMT lowered the CI but increased the AI in cassava. The 1045/1022 ratio reflects the CI benchmark, while the 1022/995 ratio reflects the AI benchmark [15, 34, 35]. Our XRD analyses also showed that HMT decreased the crystallinity degree in cassava, consistent with a previous report [36], where the crystalline degree (decreased by HMT) was attributed to the lower amylopectin compounds and structural changes in crystalline type A–B. The A-type structure was indicated by the crystalline arrangement of amylopectin chains [28]. The A-type crystalline structure displayed firm diffraction peaks of 2Ɵ = 15° and 23°, while the crystalline structure of type B displayed medium diffraction peaks at 15° and 23° [35, 37].

The HMT treatment increased RS content in cassava and modified starch digestion in ruminants *in vitro*. Heat-moisture treatment lowered DMD levels in the rumen without affecting post-rumen DMD and DM digestibility levels. Other rumen digestibility indicators, such as gas production and VFAs, were also decreased. Rumen gas kinetic calculations showed that *B* was lowered by HMT. Consistent with the previous studies [38, 39], it was observed that the increased RS supply to the rumen inhibited rumen DMD starch degradation. However, total starch digestion was not negatively affected. Thus, adding RS appeared to shift starch digestion sites from the rumen to post-ruminal organs, such as the small and large intestines, and compensates for inhibited RS fermentation from the rumen. Heat-moisture treatment is a physical modification that increases RS content by increasing retrograde starch from cooled gelatinized starch [5]. This retrograde starch resists amylase activity [8, 22]. Resistant starch resists enzymatic degradation due to α-1,4 linked glucose (amylose) in crystalline lattices [40].

Interestingly, gas production in HMT samples in the first 6 h was increased and possibly attributed to the high reducing sugar and VRDS content, which were released by high HMT temperatures. Reducing sugar in the rumen is efficiently digested and rapidly fermented [41–44]. Therefore, an increase in the reduced sugar content might increase gas production in the rumen *in vitro*.

CH_4_ inhibition by HMT was observed at 12 h, but no significant differences in production were identified at 24 h and 48 h. This finding may be related to VRDS, RDS, and reducing sugar content in HMT cassava. These compounds are highly degradable starches, as indicated by increased gas production in the first 6 h. Starches are degraded by amylolytic bacteria, causing higher propionate production and lowered H_2_ levels which inhibit CH_4_ production. Amylolytic bacteria produce one molecule of propionate by using two molecules of hydrogen during the fermentation process of starch in the rumen [43]. Meanwhile, hydrogenotrophic methanogenesis pathways in the rumen use CO2 and H2 to produce CH4. Therefore, higher content in VRDS, RDS, and reduced sugar content might lower CH4 production. However, even though significant gas inhibition was identified at 24 h and 48 h and reduced propionate levels at 48 h, CH_4_ production did not significantly differ in HMT cassava. This phenomenon was related to methanogen populations, which were not significantly affected by HMT treatment and increased acetate production. The CH_4_ production in the rumen can be affected directly by methanogen population or indirectly by nutrient digestion [18]. In this study, HMT increase acetate concentration in the rumen. Higher acetate production from carbohydrate digestion in the rumen will produce more H_2_ for methanogenesis [43]. Thus, although carbohydrate digestion was inhibited by HMT, the production of methane was not significantly affected.

HMT-treated cassava altered VFA proportions in the rumen after 48 h, where acetate and butyrate increments were observed. This observation was in contrast to a previous study that showed that starch increased propionate but decreased acetate content [40]. High RS content may have caused this finding; RS is often classified as a dietary fiber [45] due to its resistance to the enzymatic hydrolysis of α-amylase and pullulanase *in vitro*. RS degradation may be performed by fibrolytic bacteria to release acetate. This finding was supported by increasing *Bacteroides* genera populations, which mainly degrade complex plant structural carbohydrates in the rumen [46]. Total *Bacteroides* populations in the rumen are related to dietary crude fiber content [47]. It was also observed that *S. bovis* populations, a significant rumen amylolytic bacteria associated with high starch availability and lactate production in the rumen, were increased by increasing RS content in the rumen [48]. Although lactate was not measured in our study, butyrate proportions increased after 48 h. In the rumen, lactate is metabolized to butyrate by lactate-using bacteria [48, 49]. Therefore, butyrate increments were possibly related to increased relative *S. bovis* quantities in the rumen *in vitro*.

## Conclusion

Heat-moisture treatment affected cassava starch characteristics, such as increased RS, which limited digestion in the rumen. This was manifested by decreased rumen DM degradation, gas production, VFAs, and methane for 12 h, but increased *S. bovis* and *Bacteroides* levels. Thus, HMT may limit starch digestive activity in the rumen.

## Authors’ Contributions

LOP, SS, KAS, SuS, AF, WDA, RohR, YW, RoR, RF, and KGW: Contributed to the conception and design of the study, conducted the experiments, and analyzed the data. LOP, KAS, SuS, and RF: Contributed to sample preparation. LOP, KAS, SS, and KGW: Drafted the manuscript. All authors have read, reviewed, and approved the final manuscript.
